# Knowledge and Attitude towards Orthodontic Treatment among Non-Orthodontic Specialists: An Online Survey in Croatia

**DOI:** 10.3390/dj10010005

**Published:** 2022-01-03

**Authors:** Sandra Brkanović, Marina Lapter Varga, Senka Meštrović

**Affiliations:** 1 School of Dental Medicine, University of Zagreb, Gundulićeva 5, 10000 Zagreb, Croatia; 2 Department of Orthodontics, School of Dental Medicine, University of Zagreb, Gundulićeva 5, 10000 Zagreb, Croatia; lapter@sfzg.hr (M.L.V.); mestrovic@sfzg.hr (S.M.)

**Keywords:** attitude, knowledge, orthodontic treatment, specialists

## Abstract

Objectives: The aim of this study was to examine the knowledge and attitudes towards orthodontic treatment among non-orthodontic specialists. Methods: A web-based survey was formulated for non-orthodontic dental specialists to respond to statements regarding an orthodontic treatment. It contained 20 multiple-choice questions with three or more possible answers. Two hundred and fifty questionnaires were sent via email, with explanatory letters, to randomly selected non-orthodontic Croatian dental specialists. Data were assessed using IBAM SPSS 23.0. and *p* < 0.01 was considered significant. Results: The results indicate that the majority of respondents were well informed about principles and practices in orthodontics. All the respondents (100%) were aware that malocclusions can affect a patient’s facial aesthetic and masticatory function. The results also showed statistically significant differences in answers about contraindications for orthodontics therapy among different non-orthodontic specialists (*p* < 0.01). Private health practitioners were better informed about the ideal time for the first orthodontic appointment (74.2%) and that implants and periodontal problems are not contraindications for orthodontic treatment (over 70%), in comparison with public health practitioners. Conclusion: Non-orthodontic specialists in this sample exhibit encouraging awareness and knowledge of the principals and practices of orthodontic treatment. Additional improving of practitioners’ knowledge and awareness can help patients with malocclusion to decide upon orthodontic treatment at earlier stages and avoid later complications in the future.

## 1. Introduction

The aetiology of malocclusion includes several causes, but the most common consequences of malocclusion are unaesthetic facial appearance, low self-esteem, increased caries prevalence, TMD, difficulty chewing and speech, etc. [1,2,3]. Hence, a multidisciplinary approach to patient education is important for him/her to understand the need for orthodontic treatment. The patient’s decision to seek orthodontic therapy is based on several factors, while the leading reason is aesthetic nature [4]. Because appropriate and timely referral is required, it is very important for general dentists and non-orthodontic specialists to recognize the malocclusions. Research has shown that although treatment can be fully initiated by the patient, it is most often professionally initiated or influenced by referrals from general dentists, pediatric dentists, or orthodontists [5]. The extremely large influence of the dentist on the patient’s decision has also been proven [6,7]. An important variable in the reference is that most specialists focus on the problem that caused the patient to come to them, while referral of the patient to an orthodontist is expected from a general dentist or pedodontist.

In recent years, with a rising number of adult patients seeking orthodontic therapy, the time, responsibility of recording, exchanging communication and discussing options before completing a treatment plan, and consent process is increasing [8,9,10]. Therefore, cooperation between a larger number of specialists is important in informing individuals on the benefits of orthodontic treatment. This can be achieved through a multidisciplinary approach in which general dentists and other non-orthodontic specialties can play the role of orthodontic health educators, but only if they have a good knowledge and attitude about the principles and practice of orthodontic treatment. In addition, the parallel implementation of several therapies, i.e., interdisciplinary cooperation, leads to better and lasting results.

The aim of this survey was to examine the knowledge and attitudes towards orthodontic treatment among non-orthodontic specialists. Given the possible differences in fields of education, perceptions of the need for orthodontic treatment may vary depending on the dental group or specialization. Although the gold standard for assessing the needs of orthodontic treatment is the assessment of orthodontists, it is important to understand the perceptions of other dentists because they can directly and indirectly affect the use and success of orthodontic treatment. To the best of our knowledge, no similar studies have been done in Croatia or in central Europe.

## 2. Material and Methods

Ethics approval to conduct this study was granted by the ethical committee of Zagreb University, School of Dental medicine, Zagreb, Croatia (05-PA-30-XXVII-5/2021).

### 2.1. Study Design

A web-based survey was formulated for non-orthodontic dental specialists to respond to statements regarding an orthodontic treatment. The survey consisted of two sections: (1) general information such as gender, age, title, employment, and specialization and (2) attitude and awareness of principles and practices in orthodontics. It contained 20 multiple-choice questions with three or more possible answers (Appendix A). For the pilot study, the survey was emailed to five dental specialists. They were instructed to specify the presence of confusing or misleading questions. One of the specialists suggested alterations to the questionnaire. Final modifications were made according to the assessment.

Two hundred and fifty questionnaires were sent via email with explanatory letters to randomly selected Croatian dental specialists. The number of letters sent to each group of specialists was determined according to the percentage of their representation in Croatia. The letter clarified the aim of the study and that completion of the survey was entirely voluntary. Responses were not identifiable and no personal identification was requested. The estimated time for the dentist to answer the questionnaire was 7 min. A reminder email was sent out four weeks later to increase participation; therefore, responses were received over a period of eight weeks. Uncompleted questionnaires were not submitted and only the authors had access to the collected data.

### 2.2. Sample Size

A sample size of 152 dentists was taken using the following formula:

$$Ss=\frac{\left[z^{2}\times p\left(1-p)/e^{2}\right)\right]}{1+\left(\frac{z^{2}\times p\left(1-p\right)}{e^{2}N}\right)}$$

Determined values were Z-score (*z*)—1.96; anticipated proportion of specialists demonstrating to have knowledge (*p*)—0.5; margin of error (*e*)—5%; population size (*N)*—250. The size of the population was defined by number of specialists registered in specialist societies and associations within Croatian Dental Chamber (dentists who practice dental medicine in the Republic of Croatia must be members of the Chamber). According to the formula, the minimum required sample size was 152.

The sample was further divided according to the subject’s field of specialization and place of employment.

### 2.3. Statistical Analysis

The statistical analysis was performed using SPSS (version 23.0; IBM, Armonk, NY, USA) and the results were presented in frequency tables. Comparisons between groups were carried out using the Chi-square test of independence, for 3-by-7 and 2-by-3 cross tables; when necessary, the Fisher exact test was applied. The results were evaluated against a 95% confidence interval and *p* < 0.01 was considered significant.

## 3. Results

Of the 250 surveys, 158 were returned, for a response rate of 63%. A higher female predominance was found, accounting for 61.4% of the responders. The demographic characteristics of the survey subjects also showed that the largest number of responders were aged between 35 and 49 years old (57.6%), followed by the number of responders aged between 50 and 65 years old, and the smallest number of specialists was between 27 and 34 years old. Regarding the type of specialist, responders were divided into seven groups: prosthodontists, oral surgeons, endodontists, periodontists, family dentists, pedodontists, and doctors of oral medicine. The greater part of the responders was employed in public dental health (57.6%), 41.8% were employed in private dental health, and 1 responder did not practice dentistry (Table 1).

Table 2 shows the results from the questionnaire about attitude and awareness of principles and practices in orthodontics. The results indicate that the majority of respondents were well informed about principles and practices in orthodontics. All the respondents (100%) were aware that malocclusions can affect a patient’s facial aesthetic and masticatory function.

The results showed statistically significant differences in answers about contraindications for orthodontics therapy among different non-orthodontic specialists (questions 12 and 13, *p* < 0.01) (Table 3). Overall, orthodontic treatment was denied for periodontal patients by a large proportion of dentists (50.6%).

Lastly, the answers were compared between public health and private health practitioners. Comparison showed statistically significant differences in a few questions (Table 4, *p* < 0.01). Private health practitioners were better informed about the ideal time for the first orthodontic appointment (74.2%) and that implants and periodontal problems are not contraindications for orthodontic treatment (over 70%). This indicates that private health practitioners had more knowledge about interdisciplinary collaboration. Specialists in public health responded in a higher percentage that patients with bad oral habits (thumb sucking, tongue thrusting, or mouth breathing) should without delay be referred to an orthodontist (37.4%).

## 4. Discussion

Malocclusion is the third most common disease in dentistry, after dental caries and periodontal diseases [11]. Negative physical, social, and psychological impacts of malocclusions on patients make it a serious worldwide public health problem [12]. Therefore, timely and appropriate therapy is crucial for several reasons. It prevents the occurrence of severe disorders, creating the preconditions for the normal development of the orofacial system, and in this way minimizes the negative impacts of malocclusion. In addition, it very often reduces the cost of treatment because cases of neglected malocclusions require complex and expensive therapy. Precisely because of this, in order to achieve excellence, modern dentistry is based on interdisciplinary collaboration. Furthermore, studies showed that the level of a patient’s dental health knowledge and attitude, seeking a specialist treatment, and starting timely treatment are all interlinked, and they fundamentally depend on the level of knowledge and positive attitude of the dental practitioners [13]. This survey focuses on the awareness and knowledge of specialists about orthodontic therapy as a major factor for possible collaboration with an orthodontist. It highlights a highly significant difference in the knowledge as well as the attitude of specialists towards various parts of orthodontic treatment. Unsurprisingly, the majority of respondents were women, since there are more women in the profession [14]. It was also noted that the smallest number of specialists was under 35 years old. The reason is that many young dentists are still waiting for the beginning of their specialization or its completion.

In order for therapy to begin in a timely manner, it is important to know when the first examination is needed, what the ideal time is to start therapy, and what the age limit is to begin. Studies have shown that the ideal time for the first examination is 7 years old [15], with which most respondents agreed. As expected, the largest percentage of pedodontists responded correctly. Meanwhile, specialists in oral surgery, periodontology, and prosthetics answered this question correctly only 50% of the time. It is convenient that almost all doctors were aware that age does not represent a limit for orthodontic therapy.

Children very often have bad oral habits (thumb sucking, lip sucking, lip licking, mouth breathing, nail biting, etc.) and they have a role in the etiopathogenesis of malocclusions [16]. It is important to eliminate bad oral habits as soon as possible, to ensure a functional environment adequate for physiological growth [17]. Perhaps one of the most interesting findings in this study is that most respondents believed that the bad oral habits should be eliminated before the orthodontic examination. Private health practitioners claimed this opinion in a higher percentage than public health practitioners. A study done by Tanaka et al. showed that inadequate techniques for elimination of bad oral habits can prolong removal, worsen malocclusion, and waste time that can be used to initiate therapy [18]. Orthodontists are specially educated for treating bad oral habits, including their elimination. Specialists need to be further informed about this problem, especially pedodontists and family dentists.

As previously mentioned, the number of adult patients seeking orthodontic therapy has increased in the last few decades. There are more periodontal patients and patients with implants seeking orthodontic therapy. Studies have even shown that in periodontal patients, orthodontic therapy helps treat the underlying disease of periodontitis. Tooth alignment in these patients provides additional stability to the teeth, causing the formation of new bone and strengthening the teeth, and patients have also shown an improvement in oral hygiene [19,20]. This combination can achieve several benefits: stabilization or recovery of alveolar bone height, correction of dental positions, stabilization of new positions by splints, and significant improvement in facial profiles. Orthodontic therapy is also advisable in patients with implants to improve aesthetics [21]. Unfortunately, our respondents did not show the expected knowledge in this subject domain, except for oral surgeons and periodontists who are sub-specialized in working with implants, i.e., periodontal patients. In this way, many specialists would not refer a patient with malocclusion and a periodontal problem or an orthodontic implant. Comparative evaluation of public health and private health practitioners’ answers showed that private health practitioners were more aware of the fact that dental implants and periodontal problems are not contraindications for orthodontic treatment.

In the present study, the majority (more than 95%) of the respondents agreed that malocclusions affect a patient’s facial aesthetic, mastication, and self-esteem. These findings were in accordance with previous studies, wherein they found that dentists are aware that the primary benefits of orthodontic treatment are psychosocial in nature; they also believe that it will reduce susceptibility of dental disease [22,23]. Numerous studies have confirmed that the influence of dentists strongly impacts patients’ decision to undergo orthodontic treatment [6,7,24,25,26]. It is therefore important that specialists have a clear understanding of the health gain likely to accrue from orthodontic treatment. No significant difference in responses was observed depending on the type of specialty and type of employment.

After the completion of orthodontic treatment, according to the guidelines of the British Orthodontic Society (BOS), retention is mandatory. Lately, fixed retention has been increasingly used, hence many studies have been carried out about its pros and cons. The biggest concern for fixed retainers in long-term use is whether they make it more difficult to maintain oral hygiene and cause negative effects on periodontal health. The present survey indicated that respondents have a positive attitude toward fixed retainers. The majority replied that removal of the retainers is not required. Interestingly, endodontists and oral surgeons largely responded that they should be removed after 2 or 5 years. We expected that periodontists, due to the proven increased accumulation of dental plaque and calculus, would consider that retention should be removed after a certain time.

Given that we currently live in a pandemic, it was important to examine the views of dental specialists on the association of the risk of infection with starting an orthodontic therapy during pandemic. Although new research is published every day, the prevailing opinion is that dentists have a higher risk of COVID-19 infection, but patients are not at increased risk of infection in the office [26,27]. In the present study, the majority (96%) of the respondents agreed that starting orthodontic treatment during the pandemic does not increase the risk of COVID-19 infection. This statement is supported by the previous multinational study conducted by Kamate et al., who concluded that dental practitioners obtain good knowledge and practice scores [28]. It is imperative for the dentist to adopt infection prevention and control strategies, follow the CDC and WHO guidelines in their clinics, and keep treating patients.

## 5. Conclusions

Within the limitations of the study, it was concluded that most of the respondents were aware of principles and practices of orthodontic treatment. Non-orthodontic specialists in the private sector showed better knowledge about time of referral and indication for orthodontic treatment in comparison with public health practitioners.

## Figures and Tables

**Table 1:** The characteristics of the non-orthodontic specialists.

	*n* (Number)	% (Percent)
Gender		
Female	97	61.4
Male	61	38.6
Age (years)		
27–34	30	19
35–49	91	57.6
50–65	37	23.4
Specialists		
Oral surgeon	32	20.3
Prosthodontist	32	20.3
Endodontist	26	16.5
Periodontist	20	12.7
Family dentist	20	12.7
Pedodontist	15	9.5
Oral medicine	13	8.2
You are a dental practitioner in:		
Private health practice	91	57.6
Public health practice	66	41.8
I do not practice dentistry	1	0.6

**Table 2:** Results from the questionnaire about attitude and awareness of principles and practices in orthodontics.

	*n*	%
Ideal time for first orthodontic appointment		
Age of 7 years	92	58.2
After erupting first permanent premolar	48	30.4
After erupting second permanent molar	18	11.4
Ideal time for starting orthodontic therapy		
6–8 years old	9	5.7
13–16 years old	31	19.6
Depends on dental anomalies	118	74.7
Upper age limit for orthodontic therapy		
40 years old	5	3.2
50 years old	4	2.5
Doesn’t exist	149	94.3
Patients with diastema in primary dentition should be referred to orthodontist		
Yes	8	5.1
No	142	69.9
I don’t know	8	5.1
Patients with bad oral habit (thumb sucking, tongue thrusting, or mouth breathing) should		
Be directly referred to an orthodontist	46	29.1
Stop bad habits and then be referred to an orthodontist	112	70.9
Wait for permanent dentition before any orthodontic treatment	0	0
Malocclusion affects facial aesthetic		
Yes	158	100
No	0	0
I don’t know	0	0
Malocclusion affects a masticatory function		
Yes	158	100
No	0	0
I don’t know	0	0
Orthodontic therapy is contraindicated in patients with implants		
Yes	16	10.1
No	96	60.6
I don’t know	46	29.1
Orthodontic therapy is contraindicated in periodontal patients		
Yes	60	38.0
No	80	50.6
I don’t know	18	11.4
Malocclusion may impede oral hygiene		
Yes	115	72.8
No	36	22.8
I don’t know	7	4.4
Untreated cavity can cause malocclusion		
Yes	133	84.2
No	18	11.4
I don’t know	7	4.4
Orthodontic treatment always requires tooth extraction		
Yes	1	0.6
No	157	99.4
I don’t know	0	0
Retention is required after orthodontic treatment		
Yes	148	93.7
No	9	5.7
I don’t know	1	0.6
Fixed retainer removal		
After 2 years	23	14.6
After 5 years	16	10.1
It is not required	119	75.3
Orthodontic treatment outcomes affect the patient’s self-esteem		
Yes	157	99.4
No	1	0.6
I don’t know	0	0
Starting orthodontic treatment during pandemic increases risk of COVID-19 infection		
Yes	5	3.2
No	140	86.6
I don’t know	13	8.2

**Table 3:** Comparison of answers of different non-orthodontic specialties.

Question 12 (Orthodontic Therapy is Contraindicated in Patients with Implants)									Question 13 (Orthodontic Therapy is Contraindicated in Periodontal Patients)							
	Yes		No		I Don’t Know		*p*-Value	X^2^	Yes		No		I Don’t Know		*p*-Value	X^2^
	*n*	%	*n*	%	*n*	%	*p* < 0.001 *	48.52	*n*	%	*n*	%	*n*	%	*p* = 0.007 *	*27.36*
Oral surgeon	0	0	31	96.8	1	3.2			10	31.2	18	56.2	4	12.6		
Prosthodontist	3	9.4	20	62.5	9	28.1			17	53.1	12	37.5	3	9.4		
Endodontist	3	11.5	5	19.3	18	69.2			12	46.1	10	38.5	4	15.4		
Periodontist	1	5	16	80	3	15			0	0	19	95	1	5		
Family dentist	6	30	8	40	6	30			11	55	9	45	0	0		
Pedodontist	2	13.4	8	53.3	5	33.3			6	40	7	46.7	2	13.3		
Oral medicine	1	7.8	8	61.5	4	30.7			4	30.8	5	38.5	4	30.7		

* Difference was statistically significant.

**Table 4:** Comparison of answers of public health and private health practitioners.

	Public Health Practitioners		Private Health Practitioners			
	*n*	%	*n*	%	*p*-Value	X^2^
Ideal time for first orthodontic appointment					*p* = 0.003 *	11.64
Age of 7 years	43	47.2	49	74.2		
After erupting first permanent premolar	34	37.4	13	19.7		
After erupting second permanent molar	14	15.4	4	6.1		
Patient with bad oral habit (thumb sucking, tongue thrust, or mouth breathing) should					*p* = 0.009 *	9.79
Without delay be referred to orthodontic appointment	34	37.4	12	18.1		
Stop bad habits and then be referred to orthodontist	57	62.6	54	81.9		
Wait for permanent dentition before any orthodontic treatment	0	0	0	0		
Orthodontic therapy is contraindicated in patients with implants					*p* < 0.001 *	28.23
Yes	11	12.1	5	7.6		
No	39	42.8	56	84.8		
I don’t know	41	45.1	5	7.6		
Orthodontic therapy is contraindicated in periodontal patients					*p* < 0.001 *	26.62
Yes	38	41.7	15	22.8		
No	39	42.8	47	71.2		
I don’t know	14	15.5	4	6		

* Difference was statistically significant.

## Data Availability

Not applicable.

## Appendix A. Study Questionnaire

**Informed consent:**

Do you consent to take part in this study?

- Yes
- No

**Section 1**

1. Gender:
  - Female
  - Male

2. Age:
  - 27–34 years old
  - 35–49 years old
  - 50–65 years old

3. Specialist:
  - Oral surgeon
  - Prosthodontist
  - Endodontist
  - Periodontist
  - Pedodontist
  - Doctor of oral medicine
  - Family dentist

4. Employment:
  - In public health practice
  - In private health practice
  - I don’t practice dentistry

5. When is an ideal time for the first orthodontic appointment?
  - Age of 7 years
  - After erupting first permanent premolar
  - After erupting second permanent molar

6. When is an ideal time for starting orthodontic therapy?
  1. 6–8 years old
  2. 13–16 years old
  3. Depends on dental anomalies

7. What is an upper age limit for starting an orthodontic therapy?
  - 40 years old
  - 50 years old
  - It doesn’t exist

8. Would you refer patients with diastema in primary dentition to an orthodontist?
  - Yes
  - No
  - I don’t know

9. Patients with bad oral habits (thumb sucking, tongue thrust, or mouth breathing) should:
  - Be directly referred to an orthodontist
  - Stop bad habits and then be referred to an orthodontist
  - Wait for permanent dentition before any orthodontic treatment

10. Does malocclusion affect facial aesthetic?
  - Yes
  - No
  - I don’t know

11. Does malocclusion affect a masticatory function?
  - Yes
  - No
  - I don’t know

12. Is orthodontic therapy contraindicated in patients with implants?
  - Yes
  - No
  - I don’t know

13. Is orthodontic therapy contraindicated in periodontal patients?
  - Yes
  - No
  - I don’t know

14. May malocclusions impede oral hygiene?
  - Yes
  - No
  - I don’t know

15. Could an untreated cavity cause malocclusion?
  - Yes
  - No
  - I don’t know

16. Does orthodontic treatment always require tooth extraction?
  - Yes
  - No
  - I don’t know

17. Is retention required after orthodontic treatment?
  - Yes
  - No
  - I don’t know

18. When is recommended fixed retainer removal?
  - After 2 years
  - After 5 years
  - It is not required

19. Does orthodontic treatment outcome affect the patient’s self-esteem?
  - Yes
  - No
  - I don’t know

20. Does starting orthodontic treatment during pandemic increase risk of COVID-19 infection?
  - Yes
  - No
  - I don’t know
